# Supplementary material for: Single-photon emission from single-electron transport in a SAW-driven lateral light-emitting diode
Source: Nat Commun. 2020 Feb 14;11:917. doi: 10.1038/s41467-020-14560-1 (PMC7021712; doi:10.1038/s41467-020-14560-1)
Supplement: Supplementary file 1 — Supplementary Information [file 41467_2020_14560_MOESM1_ESM.pdf]

# Supplementary Information for : Single-photon emission from single-electron transport in a SAW-driven lateral light-emitting diode

Hsiao *et al.*

### Supplementary Note 1: Exciton Energy

Energy of the neutral exciton in this 15 nm-quantum-well structure can be characterised by photoluminescence (PL). Neutral excitons are created in the quantum well by optical excitation using a 645 nm laser diode. Supplementary Figure 1 shows the PL spectrum of the 15 nm GaAs quantum well, taken in the *n*-*i*-*p*-junction region at 1.5 K. The main 1.530 eV peak (FWHM $\sim$ 2 meV) is due to the excitonic transition of electrons from the conduction band to the first heavy-hole subband. The peak in the spectrum was also observed in EL signals from multiple lateral *n*-*i*-*p* junctions based on the same 15 nm quantum well, indicating that the EL signals are dominated by the neutral excitons.

### Supplementary Note 2: Fitting for Time-resolved EL Data

A theoretical function describing the SAW-driven EL can be established to fit the time-resolved measurement results. Because the SAW-driven charge transport gives rise to a sudden increase in the density of electrons in the region of holes, and the density of electrons then decays due to the electron-hole recombination, the basic function for each peak in a time-correlated histogram will be

$$\begin{aligned} f(t, t_0, A_{\mathrm{EL}}, \tau) &= 0 && \text{when} \quad t < t_0\,; \\ &= A_{\mathrm{EL}} \cdot \exp((t - t_0)/\tau) && \text{when} \quad t \geq t_0\,, \end{aligned} \tag{1}$$

where $t$ is time, $t_0$ is the arrival time of the SAW potential minimum leading to the peak, $A_{\mathrm{EL}}$ is the amplitude (peak value), and $\tau$ is the carrier lifetime of SAW-driven electrons. Since the EL is driven by a series of SAW potential minima, the overall function can be written as

$$F(t, t_0, A_{\mathrm{EL}}, \tau, t_{\mathrm{SAW}}) = \sum_{i=0,1,2,3\ldots} f(t, t_0 + i \cdot t_{\mathrm{SAW}}, A_{\mathrm{EL}}, \tau)\,, \tag{2}$$

where $t_{\mathrm{SAW}}$ is the period of the SAW. In order to fit experimental data, temporal uncertainty (jitter) has to be incorporated into the fitting function. A source for the jitter is the response of the SPAD. Ideally, the response of a perfect SPAD should be a delta function. However, the SPAD response curve exhibits a peak with a finite FWHM of

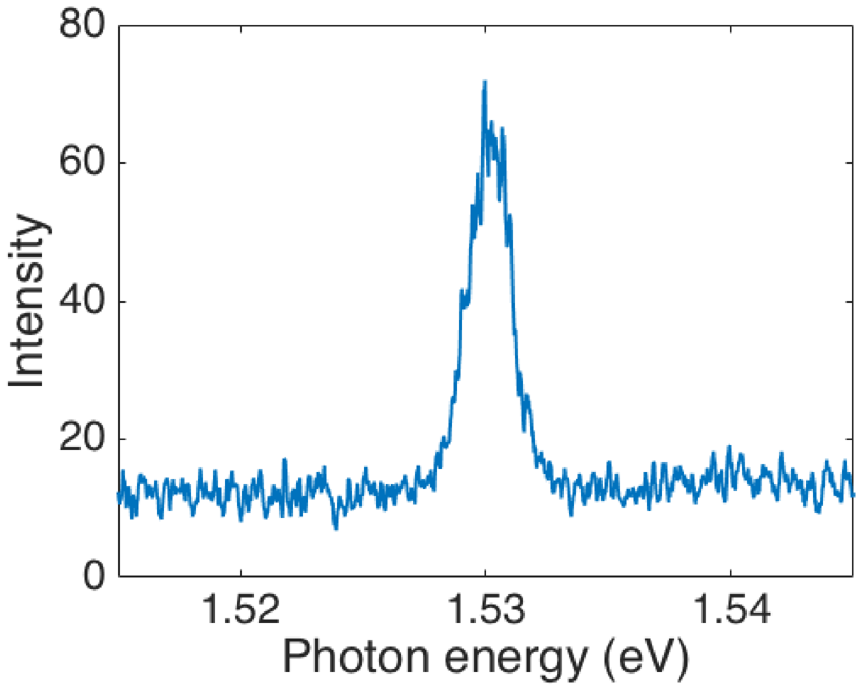


FIG. Supplementary Figure 1. Photoluminescence (PL) spectrum of the 15 nm GaAs quantum well at 1.5 K.

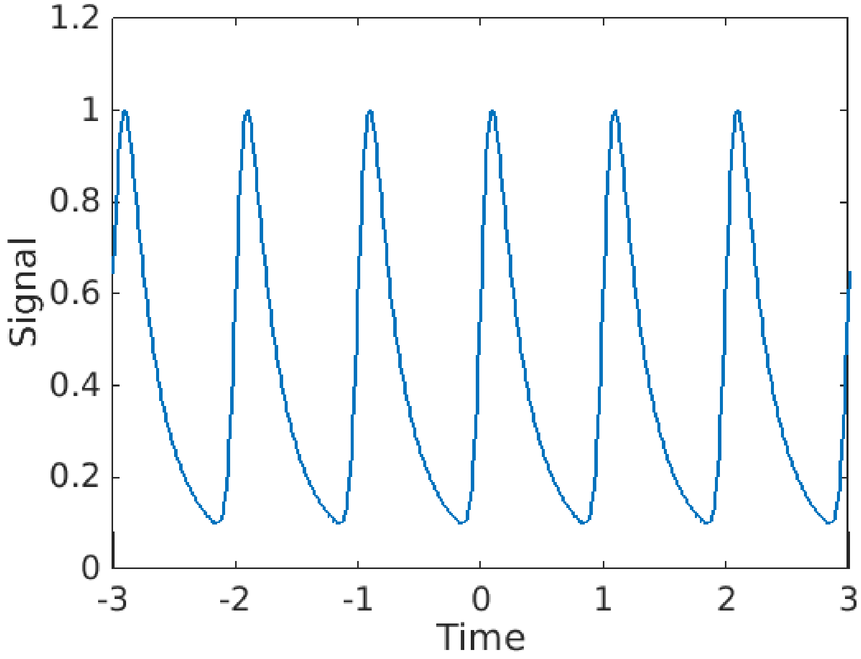


FIG. Supplementary Figure 2. Example of function $H(t)$ with $t_0 = 1$, $A_{\mathrm{EL}} = 1$, $\tau = 0.2$, $w = 0.05$, $t_{\mathrm{SAW}} = 1$, and $BG_{\mathrm{EL}} = 0$.

40 ps. This response curve works as a point spread function $PSF(t)$ so that the measured histogram will be blurred. In addition, the temporal uncertainty of SAW-driven electrons, originating from uncertainty about the position of electrons in each SAW potential minimum, can also be another source of jitter. This jitter in the SAW-driven charge transport can be expressed as a Gaussian function

$$g(t, w) = \frac{1}{w\sqrt{2\pi}} \exp\left(-\frac{1}{2}\left(\frac{t}{w}\right)^2\right), \tag{3}$$

where $w$ is the parameter related to the FWHM according to FWHM $\sim 2.3w$. Considering these sources of jitter, the time-resolved histogram $H(t)$ of the SAW-driven EL can be described as the convolution of the basic function $F$, the Gaussian function $g$, and the response curve $PSF$

$$H(t) = ((F * g) * PSF)(t) + BG_{\mathrm{EL}}, \tag{4}$$

where $BG_{\mathrm{EL}}$ is a constant for a constant background in the SAW-driven EL, which may result from long-lifetime excitons or after-pulsing of the SPADs. $H(t)$ is therefore determined by those previously mentioned parameters $t_0$, $t_{\mathrm{SAW}}$, $\tau$, $w$, $A$ and $BG_{\mathrm{EL}}$. Supplementary Figure 2 shows an example of $H(t)$. This function $H(t)$ is then used to fit the time-resolved EL data.

## Supplementary Note 3: Fitting for Autocorrelation Histogram

A function describing the autocorrelation histogram of a SAW-driven EL signal has to be established in order to fit the measured autocorrelation histogram. First, the autocorrelation $G(\Delta t)$ of a real signal $S(t)$ can be expressed as

$$G(\Delta t) = \int S(u)S(u - \Delta t)du\,. \tag{5}$$

This can be re-written as

$$\begin{aligned} G(\Delta t) &= \int S(u)S(-(\Delta t - u))du = \int S(u)S_{\mathrm{mirror}}(\Delta t - u)du \\ &= (S * S_{\mathrm{mirror}})(\Delta t)\,, \end{aligned} \tag{6}$$

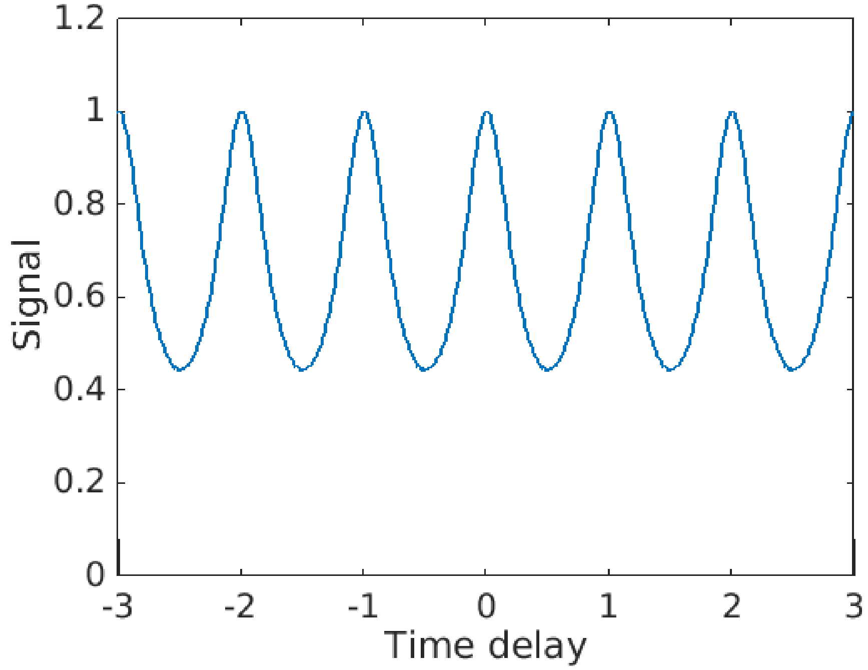


FIG. Supplementary Figure 3. Example of $G(\Delta t)$, which is the convolution of $H(t)$ in Supplementary Figure 2 and its mirror image $H_{\mathrm{mirror}}(t)$.

where $S_{\mathrm{mirror}}(t)$ is the mirror image of $S(t)$ so that $S_{\mathrm{mirror}}(t) = S(-t)$. So, the autocorrelation $G(\Delta t)$ can also be understood as the convolution of $S(t)$ and its mirror image $S_{\mathrm{mirror}}(t)$.

In the case of an autocorrelation histogram of the SAW-driven EL, the function $G(\Delta t)$ becomes

$$G(\Delta t) = (H * H_{\mathrm{mirror}})(\Delta t)\,. \tag{7}$$

where $H = ((F * g) * PSF) + BG_{\mathrm{EL}}$ (Eq. 4). This means the autocorrelation histogram can be obtained by the convolution of the SAW-driven EL $H(t)$ and its mirror image $H_{\mathrm{mirror}}(t)$. One such example is shown in Supplementary Figure 3. This function $G(\Delta t)$ can then be used to fit the measured autocorrelation histogram.

Because a constant background $BG_{\mathrm{EL}}$ only causes a constant background $BG_{\mathrm{g2}}$ in the autocorrelation, Eq. 7 can be re-written as

$$G(\Delta t) = (H' * H'_{\mathrm{mirror}})(\Delta t) + BG_{\mathrm{g2}}\,, \tag{8}$$

where $H'(t) = ((F * g) * PSF)(t)$ is the function for the SAW-driven EL without $BG_{\mathrm{EL}}$, and $H'_{\mathrm{mirror}}(t)$ is its mirror image.

### Supplementary Note 4: Extraction of the Second-order Correlation Function $g^{(2)}(\Delta t)$

To calculate the contribution of one specific peak to the autocorrelation histogram, a theoretical function describing the shape of each peak also has to be established. Since $F$ is the summation of a series of equally-spaced function $f$ (see Eq. 2), it can be shown that

$$\begin{aligned} G(\Delta t) &= \sum_{i=-n}^{n} (h * h_{\mathrm{mirror}})(\Delta t + i \cdot t_{\mathrm{SAW}}) + BG_{\mathrm{g2}} \\ &= \sum_{i=-n}^{n} J(\Delta t + i \cdot t_{\mathrm{SAW}}) + BG_{\mathrm{g2}}\,, \end{aligned} \tag{9}$$

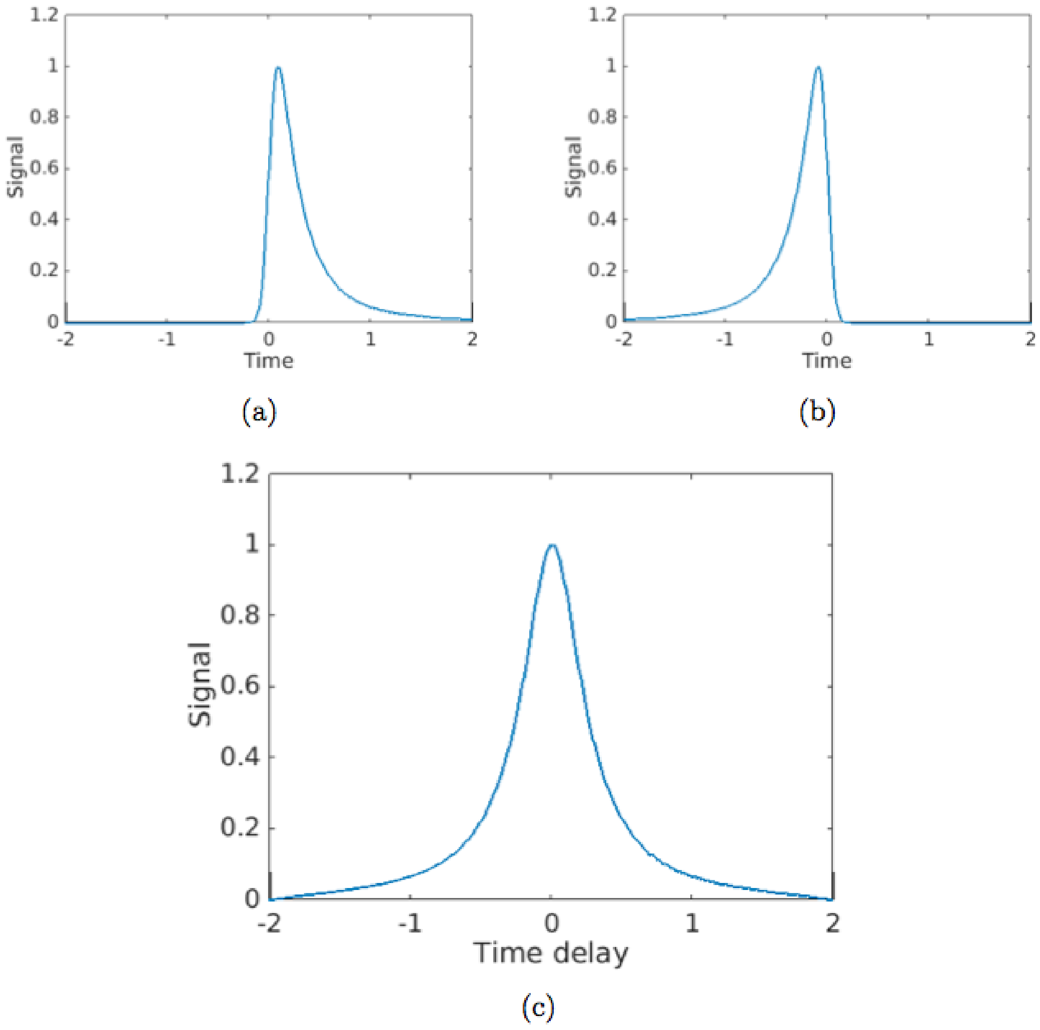


FIG. Supplementary Figure 4. (a) Example of $h(t)$ with $\tau = 0.2$, and $w = 0.05$. (b) Mirror image $h_{\mathrm{mirror}}(t)$ of $h(t)$. (c) $g_{\mathrm{auto}}(\Delta t)$, which is the convolution of $h(t)$ and $h_{\mathrm{mirror}}(t)$.

where $n \to \infty$ is the number of EL peaks, and

$$h(t) = ((f * g) * PSF)(t)\,. \tag{10}$$

$h(t)$ can be understood as the EL signal from a SAW minimum. So, the autocorrelation histogram can be seen as the summation of a series of equally-spaced functions $J(\Delta t) = (h * h_{\mathrm{mirror}})(\Delta t)$. Supplementary Figure 4(a) shows an example of $h(t)$ with $\tau = 0.2$, and $w = 0.05$ while Supplementary Figure 4(b) shows the mirror image $h_{\mathrm{mirror}}(t)$. Their convolution $J(\Delta t)$ is plotted in Supplementary Figure 4(c). This single peak can be understood as the actual shape of individual peaks in Supplementary Figure 3, which means that the contribution from a specific peak can be individually evaluated even though there is significant overlap between these peaks. So, if the theoretical function $J(\Delta t)$ for SAW-driven EL is known, the real signal from a suppressed peak, such as that in Fig. 3(a) in the main text, can be estimated more accurately.

From the fit for the averaged histogram in Fig. 3(b) in the main text, the shape of each peak is determined by $J(\Delta t)$ with $\tau = 99.6\,\mathrm{ps}$, and $w = 33\,\mathrm{ps}$, and $BG_{\mathrm{g2}} = 2.79$. It can be assumed that each peak has the same shape but different peak amplitude due to the statistical sample variance. These peaks at $\Delta t = \Delta t_{\mathrm{(i)}}$ have different amplitudes $A_{\mathrm{g2(i)}}$, which are proportional to $g^{(2)}(\Delta t_{\mathrm{(i)}})$. A modified autocorrelation function reflecting the variance can be expressed as

$$G'(\Delta t) = \sum_{i=-\infty}^{\infty} A_{\mathrm{g2(i)}} \cdot J(\Delta t + i \cdot t_{\mathrm{SAW}}) + BG_{\mathrm{g2}}\,. \tag{11}$$

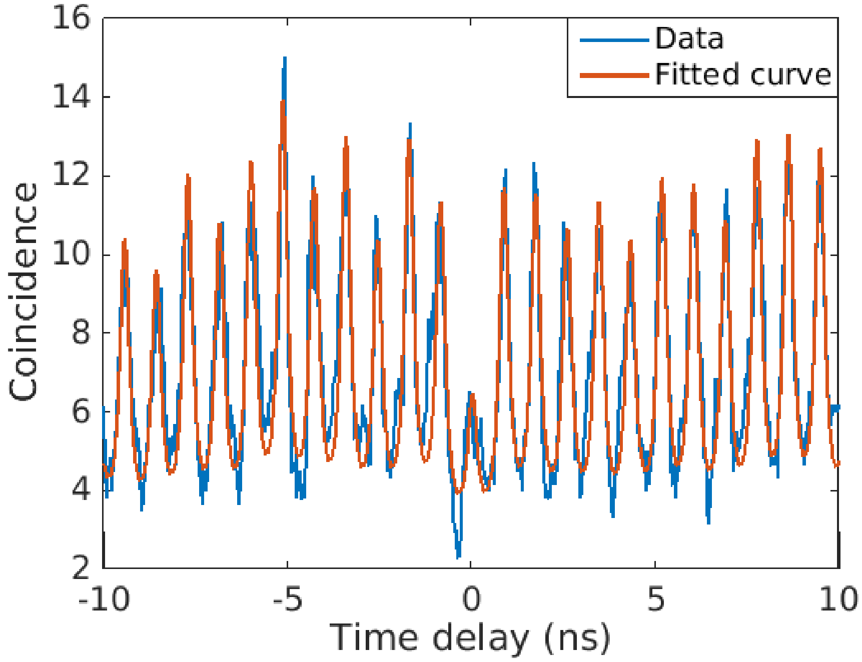


FIG. Supplementary Figure 5. Autocorrelation histogram in Fig. 3(a) in the main text and the best-fit curve using Eq. 11.

In order to evaluate $g^{(2)}(\Delta t)$, $A_{\mathrm{g2(i)}}$ at individual peaks have to be obtained by fitting the autocorrelation histogram to $G'(\Delta t)$ with $\tau = 99.6\,\mathrm{ps}$, $w = 33\,\mathrm{ps}$, and $BG_{\mathrm{g2}} = 2.79$. Supplementary Figure 5 shows the data from Fig. 3(a) in the main text, along with the best-fit curve for the suppressed peak and the nearest 22 peaks.

The fitting process assigns to each peak an amplitude $A_{\mathrm{g2(i)}}$. The second-order correlation function $g^{(2)}(\Delta t)$ is then obtained from the normalised $A_{\mathrm{g2(i)}}$ as a function of time delay, as shown in Fig. 4(a) in the main text.

## Supplementary Note 5: Estimate of the Probability Distribution

Because the number of electrons carried in a SAW minimum can be 0, 1, 2, 3 and so on, the probability $P_n$ of having $n$ electrons will follow a probability distribution. Since the average electron number $N_{\mathrm{avg}}$ in each SAW minimum is 0.89, the probability of carrying more than four electrons in a SAW minimum should be negligible. Hence, the wave function $|\psi\rangle$ for the electrons in a SAW minimum can be expressed as a superposition state

$$|\psi\rangle = \sqrt{P_0}|0\rangle + \sqrt{P_1}|1\rangle + \sqrt{P_2}|2\rangle + \sqrt{P_3}|3\rangle \,, \tag{12}$$

with

$$\sum_i P_i = 1 \text{ and } 0 \leq P_i \leq 1 \,, \tag{13}$$

where $|i\rangle$ is the $i$-electron state in the Fock basis, and $P_i$ is the probability of measuring $i$ electrons. The SAW-driven EL signal due to this electron-number probability distribution $\{P_i\}$ then leads to the second-order correlation function $g^{(2)}(0) = 0.39$. Hence, the probability distribution $\{P_i\}$ in $|\psi\rangle$ manifests $N_{\mathrm{avg}} = 0.89$ and $g^{(2)}(0) = 0.39$. $N_{\mathrm{avg}}$ and $g^{(2)}(0)$ can be expressed quantum mechanically as

$$N_{\mathrm{avg}} = \langle\psi|\hat{a}^\dagger\hat{a}|\psi\rangle = 0.89 \,, \tag{14}$$

and

$$g^{(2)}(0) = \frac{\langle\psi|\hat{a}^\dagger\hat{a}^\dagger\hat{a}\hat{a}|\psi\rangle}{\langle\psi|\hat{a}^\dagger\hat{a}|\psi\rangle^2} = 0.39 \,, \tag{15}$$

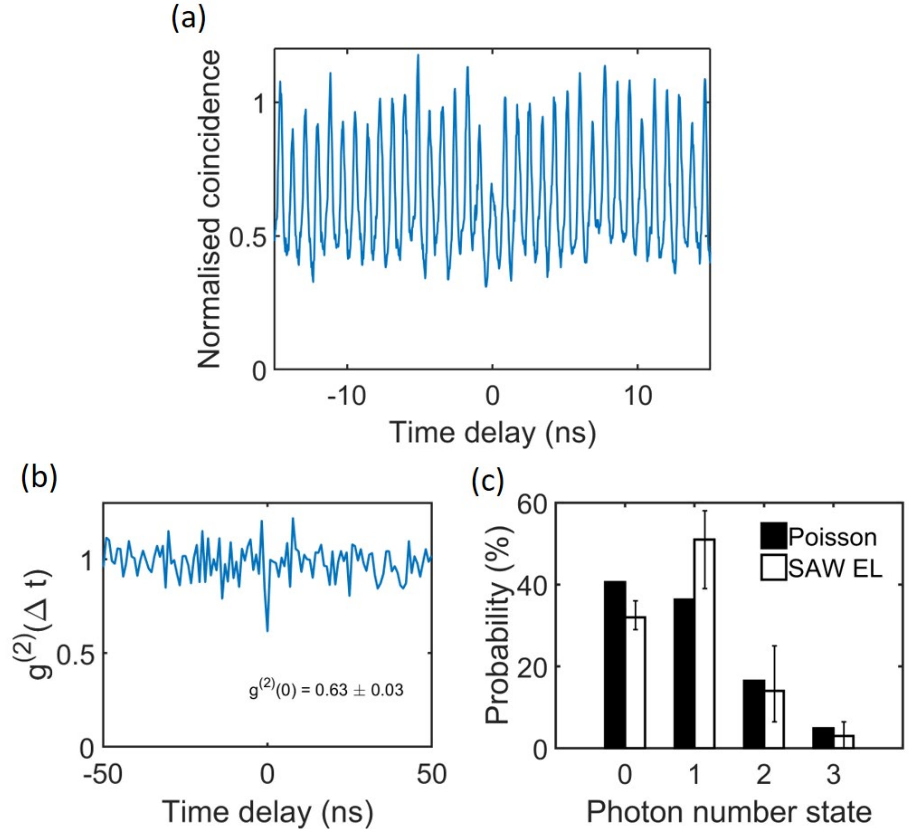


FIG. Supplementary Figure 6. Analysis of the raw data. (a) Normalised autocorrelation histogram. (b) The second order correlation function $g^{(2)}(\Delta t)$. (c) Estimated probability distribution of photon-number states compared with the probability distribution in a Poissonian light source.

where $\hat{a}$ and $\hat{a}^\dagger$ represent the annihilation and creation operators. Since $N_{\text{avg}} = 0.89 < 1$, it should be safe to assume that a SAW minimum is less likely to contain two electrons than one electron, and is even more unlikely to contain three electrons. It seems likely, therefore, that

$$\frac{P_2}{P_1} \geq \frac{P_3}{P_2}\,. \tag{16}$$

If so, taking the equality $\frac{P_2}{P_1} = \frac{P_3}{P_2}$, and hence a larger value of $P_3$, and using Equations 13 to 15, a probability distribution $\{P_0, P_1, P_2, P_3\} = \{0.25, 0.63, 0.10, 0.02\}$ can be obtained. This gives $P_1/(P_1 + P_{\text{multi}}) = P_1/(P_1 + P_2 + P_3) = 0.84$. Alternatively, suppose that $P_3 = P_2$, giving the highest possible value of $P_3$. Then $P_1 = 0.7$, which is even higher and gives $P_1/(P_1 + P_{\text{multi}}) = 0.9$ ($\{P_0, P_1, P_2, P_3\} = \{0.22, 0.7, 0.04, 0.04\}$). If, instead, $P_3 = 0$, then $P_1 = 0.58$, which gives $P_1/(P_1 + P_{\text{multi}}) = 0.79$ ($\{P_0, P_1, P_2, P_3\} = \{0.27, 0.58, 0.15, 0\}$). Hence, the estimated probability distribution is $\{0.25 \pm 0.03, 0.63 \pm 0.07, 0.10 \pm 0.06, 0.02 \pm 0.02\}$.

### Supplementary Note 6: Analysis for the raw data

We plot the raw autocorrelation histogram in Supplementary Figure 6 (a) and analyse the raw data using the methods described in S4 and S5. The extracted $g^{(2)}(\Delta t)$ is shown in Supplementary Figure 6 (b). Since some of the coincidences happened outside the single-electron regime, the raw $g^{(2)}(0) = 0.63 \pm 0.03$ is higher than the single-photon criterion 0.5. However, even without post-selection, the device still produced photons with sub-Poissonian statistics during the measurement period of 54 hours. The estimated probability distribution for the raw data is $\{0.32, 0.51, 0.14, 0.03\}$ if $\frac{P_2}{P_1} = \frac{P_3}{P_2}$, $\{0.29, 0.58, 0.065, 0.065\}$ if $P_3 = P_2$, and $\{0.36, 0.39, 0.25, 0\}$ if $P_3 = 0$.

## Supplementary Note 7: Modelling of SAW-driven Charge Transport

In the main text, the non-zero probability of multiple occupation, $P_{\text{multi}} \equiv P_2 + P_3 = 0.08$–$0.15$ when $N_{\text{avg}} = 0.89$, means that a SAW minimum may sometimes contain more than one electron and hence cause unwanted multi-photon emission, or carry no electron so that the electron-to-photon conversion does not happen. In order to understand qualitatively how to enhance the single-electron probability $P_1$, we have built a simplified model for the SAW-driven charge transport. To build the model, the first step is to calculate the electrostatic potential of the SAW-driven *n-i-p* junction with S-D bias and gate voltages similar to the experiment. The blue curve in Supplementary Figure 7 shows the conduction band along the SAW-propagation direction (the $x$ axis), where the source (electron) bias $V_{\text{source}} = -0.8\,\text{V}$, the drain (hole) bias $V_{\text{drain}} = 0.65\,\text{V}$, and the side-gate voltage $V_{\text{SiG}} = -0.4\,\text{V}$. This calculation of the electrostatic potential, based on the real device geometry, was carried out using the partial differential equation solver Nextnano [1, 2]. As can be seen, the potential difference between the region of electrons (left) and the region of holes (right) is about 80 meV, so electrons cannot overcome this potential difference and recombine with holes unless a SAW drags these electrons to the region of holes. The red curve in Supplementary Figure 7 shows the calculated conduction band with superposition of a SAW potential (amplitude $A_{\text{SAW}} = 20\,\text{meV}$). The SAW potential minima work as dynamic quantum dots, trapping the electrons and transporting them to the region of holes. During the transportation of electrons, some of them will tunnel out of the potential minima if the electrochemical potential $u_{(\text{SAWdot})}$ of the dynamic quantum dots is close to or higher than the quantum-dot barrier height $V_0$ created by the SAW. The probability of tunnelling in one attempt can be described by the transmission probability of non-interacting electrons through a saddle-point potential [3, 4]

$$T = \frac{1}{1 + e^{-\pi\epsilon_n}} \tag{17}$$

with

$$\epsilon_n = 2[u_{(\text{SAWdot})} - \hbar\omega_y(n_y + \frac{1}{2}) - V_0)]/\hbar\omega_{\text{Barrier}}\,, \tag{18}$$

where $\omega_{\text{Barrier}}$ is the curvature of the quantum-dot barrier expressed in frequency, $\omega_y$ is the curvature of the potential perpendicular to the SAW-propagation direction (the $y$ axis), which is related to the electrostatic potential of the etched 1D channel, and $n_y$ is the quantum number of the wave function on the $y$ axis. The 2D potential of a SAW-created quantum dot can be approximated as an anisotropic 2D harmonic oscillator with two different frequencies along the $x$ and $y$ axes

$$V(x,y) = \frac{1}{2}m{\omega_x}^2x^2 + \frac{1}{2}m{\omega_x}^2y^2 \tag{19}$$

with energy states

$$E(n_x,n_y) = \left(\frac{1}{2} + n_y\right)\hbar\omega_x + \left(\frac{1}{2} + n_y\right)\hbar\omega_y\,, \tag{20}$$

where $\omega_x = \omega_{\text{SAW}}$ (the curvature at the bottom of the SAW minimum) and $n_x$ is the quantum number of the wave function on the $x$ axis. Assuming (1) $\omega_x > \omega_y$ (which is true for the simulation result shown later) (2) the charging energy $E_\text{c}$ for adding one electron is dominated by the patterned gates rather than electron-electron interactions, and (3) spin-spin interactions can be ignored, then the electrochemical potential $u_{(\text{SAWdot})}(N)$ for the first six electrons in the dot can be written as [5]

$$\begin{aligned}
u_{(\text{SAWdot})}(1) &= E_\text{c} + E(0,0)\\
u_{(\text{SAWdot})}(2) &= 2E_\text{c} + E(0,0)\\
u_{(\text{SAWdot})}(3) &= 3E_\text{c} + E(0,1)\\
u_{(\text{SAWdot})}(4) &= 4E_\text{c} + E(0,1)\\
u_{(\text{SAWdot})}(5) &= 5E_\text{c} + E(1,0)\\
u_{(\text{SAWdot})}(6) &= 6E_\text{c} + E(1,0)\,.
\end{aligned} \tag{21}$$

Therefore, the tunnelling probabilities of the first $N$ electrons in the SAW-created dot (with a given SAW amplitude and at a given dot position during transport) can be calculated using Eq. 17 once $\omega_{\text{Barrier}}$, $\omega_{\text{SAW}}$, $\omega_y$, and $V_0$ are

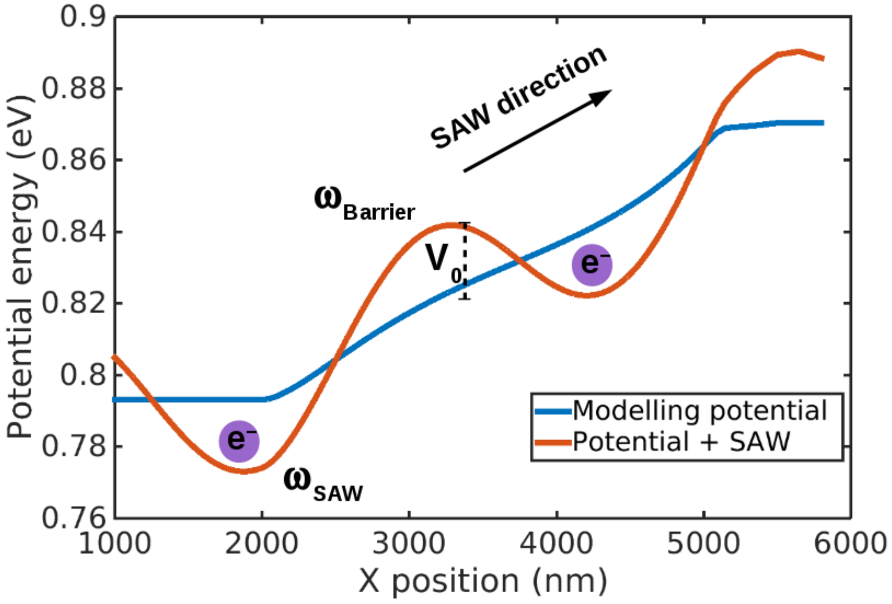


FIG. Supplementary Figure 7. Electrostatic potential of the conduction band (blue curve) of a SAW-driven *n-i-p* junction, calculated using Nextnano. The red curve shows the electrostatic potential superposed by a SAW potential with SAW amplitude of 20 meV. Electrons are trapped in the SAW minimums and dragged from the region of electrons ($x = 2000$ nm) to the region of holes ($x = 5000$ nm). Each SAW minimum works as a dynamic quantum dot (with potential height $V_0$, curvature at the top of the barrier $\omega_{\mathrm{Barrier}}$, and curvature at the bottom of the quantum dot $\omega_{\mathrm{SAW}}$).

obtained from fitting the conduction band with a SAW superposed at the corresponding phase. In Supplementary Figure 8, in order to observe the overall trend in the modelling result, a few poorly-fitted $V_0$ and $\omega_{\mathrm{Barrier}}$ are removed and interpolated. Subsequently, a probability distribution of electron numbers in the dot can be calculated by the evolution of electron population, due to tunnelling, from an initial state (e.g. 5 electrons at the beginning) in the region of electrons ($x = 2000$ nm in Supplementary Figure 7) to the final state in the region of holes ($x = 5000$ nm).

The modelling result in Supplementary Figure 8 shows the average number of electrons corresponding to the final probability distribution at $x = 5000$ nm as a function of SAW amplitude $A_{\mathrm{SAW}}$ and charging energy $E_{\mathrm{c}}$, where the initial state is 5 electrons at $x = 2000$ nm. As can be seen, at a fixed $E_{\mathrm{c}}$, when $A_{\mathrm{SAW}}$ decreases, the number of electrons also decreases from 5 to 0 due to weakening of the confinement around the SAW minimum. In addition, at a higher $E_{\mathrm{c}}$, the steps between different electron numbers are wider in terms of $A_{\mathrm{SAW}}$ and are more well-defined (with a higher contrast). This indicates that a more well-defined electron number, and thus a high-fidelity electron-to-photon conversion, may be achieved by increasing the charging energy. This requires a stronger confinement to be provided laterally by the etching and perhaps also longitudinally by the SAW potential.

## Supplementary Note 8: Future Improvements

Regarding the EL efficiency $\eta$, as electrons are carried in the SAW minima, they may pass the $p$ region without immediately recombining with holes near the junction, and eventually produce photons far away from the junction, which cannot be collected by the confocal optics. The existence of such electrons may be one of the reasons for the low $\eta = 2.5\%$. Hence, capturing electrons using etching or extra electrostatic potentials around the junction may be helpful for improving $\eta$. Having a Bragg stack beneath the quantum well and building a pillar with an optical cavity designed to be resonant with the neutral-exciton energy will also help the photons to be emitted into a desired mode, and thus increase $\eta$. Since the neutral-exciton energy in the quantum well should be reproducible between wafers, and can be characterised using photoluminescence (PL), the Bragg stack and the optical cavity can be deterministically designed for the expected photon energy, which makes the brightness enhancement more reliable than for self-assembled InGaAs QDs.

Regarding photon indistinguishability, the total spectral FWHM of the EL signal is caused by homogeneous (natural linewidth and phonon scattering) and inhomogeneous (local electric field and interface roughness) broadening. It was

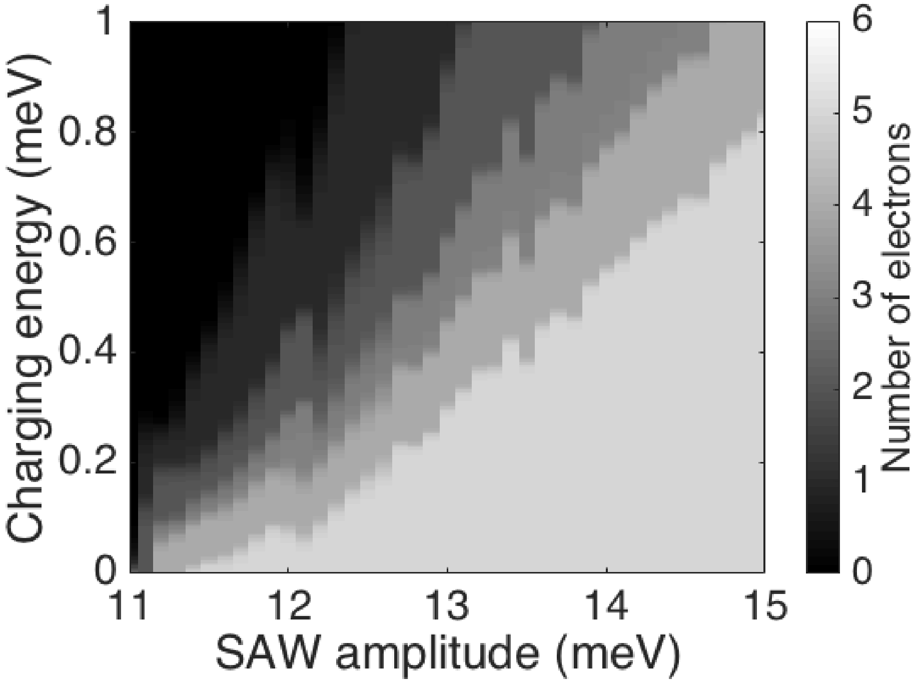


FIG. Supplementary Figure 8. Modelling result of the SAW-driven charge transport. Number of SAW-driven electrons arriving at the region of holes as a function of SAW amplitude and charging energy.

found that, in a high-quality 15 nm GaAs quantum well, the homogeneous broadening is about 100-150 $\mu$eV (FWHM) (with an acoustic-phonon broadening coefficient $\gamma \simeq 3\,\mu$eV/K), and the inhomogeneous broadening is about 100 $\mu$eV, at 8 K [6]. Therefore, if a device can be made in a high-quality quantum well with improved interface roughness (it's possible to achieve a precision of 0.01-0.1 monolayer using very slow MBE growth rate [7]), the spectral FWHM may be reduced to about 80 $\mu$eV at 1.5 K, which will improve the photon indistinguishability and increase the coherence length to $\sim$ 3.7 mm. For other applications like quantum repeaters and photonic quantum computation, two photons sent to a beam-splitter are required to remain in phase for the duration of the overlapping photon wavepackets in order to maximise photon-photon interference. For two photons with an energy difference $\Delta E_{\mathrm{ph}}$ and a wavepacket size $\Delta t_{\mathrm{ph}}$ ($\sim$ carrier lifetime $\tau$) in the time domain, the requirement can be expressed as $\frac{\Delta E_{\mathrm{ph}}}{\hbar}\Delta t_{\mathrm{ph}} \leq \pi$. If $\Delta E_{\mathrm{ph}} = 80\,\mu$eV, then $\Delta t_{\mathrm{ph}}$ needs to be shorter than 25 ps, which may be achievable by increasing the recombination rate or reducing the SAW-transport jitter.

### Supplementary Note 9: Application Potential as a Single-photon Emitter

Our device is based on a scheme proposed for making a novel single-photon source. Therefore, here we also discuss the application potential of our device in quantum photonics. Currently, most high-performance single-photon sources are based on self-assembled quantum dots (QDs) [8]. Particularly, electrically-triggered QD-based sources [9–12], with second-order correlation function $g^{(2)}(0) \leq 0.1$ and repetition rate up to 2 GHz, have attracted much research attention for making scaleable and compact photonic devices. However, due to their random growth process, the location and photon energy of QDs are hard to predict. Site-controlled growth and photon-energy tuning using an electric field or strain are thus required to address the issue of QD randomness [13, 14].

The SAW-driven lateral *n-i-p* junction is fabricated using a fully deterministic lithography process in a conventional GaAs quantum well. Hence, it does not rely on the presence of a self-assembled quantum dot and can be placed at predefined locations. In addition, the photon energy is determined by the quantum-well thickenss, which can be precisely controlled by MBE. This makes the integration with optical cavities and waveguides more reliable and reproducible. It is thus promising to place multiple synchronised SAW-driven single-photon emitters in an integrated chip for electrically-driven photonic quantum networks [15–17].

On the other hand, our device structure may also be fabricated using emerging 2D materials like $WSe_2$ and $MoS_2$.

In these 2D materials, photon polarisations are coupled to the K and K' valleys in their band structures owing to the breaking of inversion symmetry [18]. Because the gate-defined junction direction and the SAW direction can both be easily reversed in our device structure, a chiral single-photon source with electrically-controlled polarisation may be achieved.

**Supplementary References**


[1] Hou, H. *et al.* Experimental verication of electrostatic boundary conditions in gate-patterned quantum devices. *Journal of Physics D: Applied Physics* **51**, 244004 (2018).

[2] Nextnano GmbH. Nextnano.

[3] Buttiker, M. Quantized transmission of a saddle-point constriction. *Physical Review B* **41**, 7906–7909 (1990).

[4] Astley, M. R. *et al.* Energy-dependent tunneling from few-electron dynamic quantum dots. *Physical Review Letters* **99**, 156802 (2007).

[5] Kouwenhoven, L. P., Austing, D. G. & Tarucha, S. Few-electron quantum dots. *Reports on Progress in Physics* **64**, 701–736 (2001).

[6] Srinivas, V., Hryniewicz, J., Chen, Y. J. & Wood, C. E. Intrinsic linewidths and radiative lifetimes of free excitons in GaAs quantum wells. *Physical Review B* **46**, 10193–10196 (1992).

[7] Dasmahapatra, P., Sexton, J., Missous, M., Shao, C. & Kelly, M. J. Thickness control of molecular beam epitaxy grown layers at the 0.01-0.1 monolayer level. *Semiconductor Science and Technology* **27**, 085007 (2012).

[8] Senellart, P., Solomon, G. & White, A. High-performance semiconductor quantum-dot single-photon sources. *Nature Nanotechnology* **12**, 1026–1039 (2017).

[9] Yuan, Z. Electrically Driven Single-Photon Source. *Science* **295**, 102–105 (2002).

[10] Hargart, F. *et al.* Electrically driven quantum dot single-photon source at 2 GHz excitation repetition rate with ultra-low emission time jitter. *Applied Physics Letters* **102**, 011126 (2013).

[11] Petruzzella, M. *et al.* Electrically driven quantum light emission in electromechanically tuneable photonic crystal cavities. *Applied Physics Letters* **111**, 251101 (2017).

[12] Müller, T. *et al.* A quantum light-emitting diode for the standard telecom window around 1,550 nm. *Nature Communications* **9**, 862 (2018).

[13] Schneider, C. *et al.* Single site-controlled In(Ga)As/GaAs quantum dots: growth, properties and device integration. *Nanotechnology* **20**, 434012 (2009).

[14] Patel, R. B. *et al.* Two-photon interference of the emission from electrically tunable remote quantum dots. *Nature Photonics* **4**, 632–635 (2010).

[15] Spring, J. B. *et al.* Boson sampling on a photonic chip. *Science* **339**, 798–801 (2013).

[16] Wang, J. *et al.* Experimental quantum Hamiltonian learning. *Nature Physics* **13**, 551–555 (2017).

[17] Sparrow, C. *et al.* Simulating the vibrational quantum dynamics of molecules using photonics. *Nature* **557**, 660–667 (2018).

[18] Mak, K. F., He, K., Shan, J. & Heinz, T. F. Control of valley polarization in monolayer MoS2by optical helicity. *Nature Nanotechnology* **7**, 494–498 (2012).
