# Supplementary material for: Single-photon emission from single-electron transport in a SAW-driven lateral light-emitting diode
Source: Nat Commun. 2020 Feb 14;11:917. doi: 10.1038/s41467-020-14560-1 (PMC7021712; doi:10.1038/s41467-020-14560-1)
Supplement: Supplementary file 3 — Description of Additional Supplementary Files [file 41467_2020_14560_MOESM3_ESM.pdf]

Title: Supplementary Movie 1

Description: Supplementary Movie 1 shows the schematic animation for our device.
